# Magnesium Improves Cardiac Function in Experimental Uremia by Altering Cardiac Elastin Protein Content

**DOI:** 10.3390/nu15061303

**Published:** 2023-03-07

**Authors:** Xoana Barros, Xenia Friesen, Vincent Mathias Brandenburg, Elisa Anamaria Liehn, Sonja Steppan, Fabian Kiessling, Rafael Kramann, Jürgen Floege, Thilo Krüger, Nadine Kaesler

**Affiliations:** 1 Division of Nephrology and Rheumatology, RWTH Aachen University Hospital, 52074 Aachen, Germany; 2 Fundació Puigvert, 08025 Barcelona, Spain; 3 Department of Cardiology and Nephrology, Rhein-Maas Klinikum Würselen, 52146 Wuerselen, Germany; 4 Institute for Molecular Medicine, University of Southern Denmark, 5230 Odense, Denmark; 5 “Victor Babes” National Institute of Pathology, 050096 Bucharest, Romania; 6 National Heart Center Singapore, Singapore 169609, Singapore; 7 Scientific Affairs, Center of Excellence Medical, Fresenius Medical Care Deutschland GmbH, 61352 Bad Homburg, Germany; 8 Institute for Experimental Molecular Imaging, RWTH Aachen, 52074 Aachen, Germany; 9 Institute of Experimental Medicine and Systems Biology, RWTH Aachen University Hospital, 52074 Aachen, Germany

**Keywords:** magnesium, chronic kidney disease, adenine nephropathy, echocardiography, cardiac function

## Abstract

Cardiovascular complications are accompanied by life-threatening complications and represent the major cause of death in patients with chronic kidney disease (CKD). Magnesium is important for the physiology of cardiac function, and its deficiency is common in CKD. In the present study, we investigated the impact of oral magnesium carbonate supplementation on cardiac function in an experimental model of CKD induced in Wistar rats by an adenine diet. Echocardiographic analyses revealed restoration of impaired left ventricular cardiac function in animals with CKD. Cardiac histology and real-time PCR confirmed a high amount of elastin protein and increased collagen III expression in CKD rats supplemented with dietary magnesium as compared with CKD controls. Both structural proteins are crucial in maintaining cardiac health and physiology. Aortic calcium content increased in CKD as compared with tissue from control animals. Magnesium supplementation numerically lowered the increases in aortic calcium content as it remained statistically unchanged, compared with controls. In summary, the present study provides evidence for an improvement in cardiovascular function and aortic wall integrity in a rat model of CKD by magnesium, as evidenced by echocardiography and histology.

## 1. Introduction

Magnesium is an essential micronutrient involved in bone mineralization, neuro-logic stimulus transmission, and muscle contraction, and serves as a cofactor for various enzymes. Both hypermagnesemia and hypomagnesemia are associated with various disease conditions.

Hypomagnesemia is common in chronic kidney disease (CKD) and is linked to higher overall mortality rates, as indicated in a prospective observational study in over 10,000 individuals with CKD [1]. A reduced serum magnesium concentration per se increases the risk for cardiovascular and all-cause mortality [2]. The latter data were obtained from a large population-based study without a specific disease-based preselection. Several mechanisms presumably contribute to the beneficial health effects of intermediate or intermediate-to-high compared with low magnesium levels. Many of these mechanisms are cardiovascular in nature. Numerous studies investigated the effects of a dietary supplementation of magnesium and found improvements in the risk for arrhythmias such as atrial fibrillation [3,4,5,6], left ventricular function [7], and heart failure [8]. These data clearly point towards a relevant role of magnesium in myocardial function. Similarly, magnesium supplementation showed a positive impact on coronary vascular disease [9,10,11,12]. Such associative data demonstrate a reciprocal correlation between the amount of cardiovascular disease and the magnesium level, especially in terms of cardiovascular calcification. 

Magnesium might have anticalcific properties. In vitro studies as well as various clinical situations have shown that magnesium levels are associated with vascular calcification, cardiovascular disease, and altered bone-mineral metabolism—the latter indirectly driving ectopic calcifications such as mineral depositions in the vascular wall [13]. Thus, it is intriguing to speculate whether magnesium-containing oral phosphate binders might exert additional pleiotropic effects in patients with CKD other than lowering serum phosphate levels. There has been a long search to identify the ideal phosphate binder that would incorporate the optimal phosphate-lowering properties with the beneficial pleiotropic effects. Magnesium supplementation in parallel with phosphate binding and excretion might be such a magic bullet in CKD patients. To date, phosphate binders are the only approved pharmacological treatment for hyperphosphatemia. Phosphate control is accomplished primarily through calcium-free phosphate binders [14]. However, any additional improvements in clinical cardiovascular outcomes still remain elusive [15,16]. Thus, it is highly desirable to find novel approaches to improve on the current therapeutic options. Numerous associations exist between serum magnesium concentrations and cardiovascular risk factors, but mechanistic insights are scarce [17]. Further unresolved issues include the optimal dosage, source, and application route of magnesium [18].

**Figure 1:**
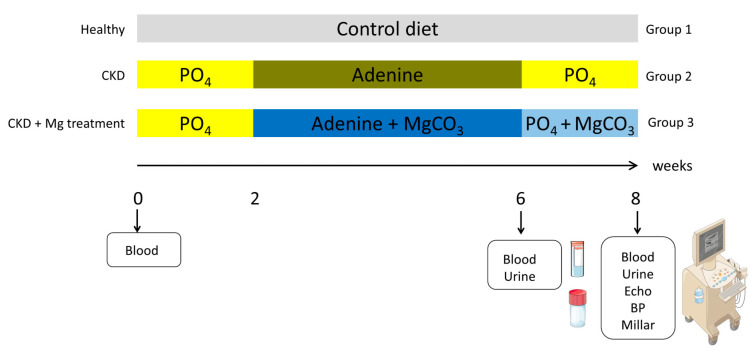
Time schedule and diet plans of adenine nephropathy in rats. Wistar rats received either a standardized control diet or an initial 2 weeks of phosphate-rich diet, followed by 4 weeks of an adenine diet—either alone or supplemented with MgCO_3_, followed by 2 weeks of a phosphate-rich diet, with or without MgCO_3_. BP: blood pressure; CKD: chronic kidney disease; MgCO_3_: magnesium carbonate; PO_4_: phosphate.

As cardiovascular complications are the major cause of death in CKD patients, we aimed to gain insight into the potential cardiac benefits of magnesium supplementation in the setting of experimental CKD (Figure 1). Rodent models of CKD offer the opportunity to examine the two major anatomic structures of the deleterious heart–kidney axis—the myocardium and the vascular wall.

Hence, we studied Wistar rats with adenine-induced nephropathy, supplemented with a magnesium-rich diet, in terms of soft tissue calcification and cardiac function. The ad libitum, adenine-containing diet was supplemented with 3.5% magnesium carbonate. We focused our analyses upon not only the structural but also the functional changes triggered by CKD and magnesium carbonate.

## 2. Materials and Methods

### 2.1. Animals 

Three groups of male Wistar rats were fed either a pelleted control diet (*n* = 8) (containing 17.8% protein and 0.7% phosphorous, V1535, Ssniff diets, Soest, Germany) (group 1) for 8 weeks or a phosphorous-enriched diet (1.03%) for 2 weeks, followed by 4 weeks of an adenine (0.9%), low-protein (2.5%) diet, either without (group 2) or with magnesium carbonate (5.82 g/kg) (group 3) *n* = 13 each). Oral adenine is metabolized to 2,8 dihydroxyadenine, leading to the formation of adenine crystals inside the kidneys and damage to the tubular structures, as well as triggering CKD-induced cardiovascular disease. Thus, this rodent model allows partial comparison to human disease [19,20]. The initial bodyweights of the rats were 340–440 g. To avoid severe weight loss, the adenine diet was stopped after 4 weeks and a high phosphorous diet (1.03%) plus magnesium carbonate was continued for 2 weeks, resulting in a total treatment period of 8 weeks (Figure 1). The prolonged high-phosphorous-containing diet is required to aggravate the cardiovascular damage on top of the kidney disease [21]. 

During the experiment, the animals were kept under standard conditions (12 h light/12 h dark) with unlimited access to food and water. The animal study was approved by the local authorities at the RWTH Aachen University Hospital and the regional administration (Landesamt für Natur, Umwelt und Verbraucherschutz, NRW, Düsseldorf, Germany). The animals were treated by trained personnel according to the ARRVIE guidelines.

### 2.2. Functional Measurements

Echocardiography was performed using a Vevo 2100 machine, equipped with a MS400 transducer (18–38 MHz) (Visual Sonics Inc., Toronto, ON, Canada) and under 2% isoflurane anesthesia on a table heated to 37 °C. Parasternal long-axis and short-axis views were recorded in B-mode. The ejection fraction and stroke volume were calculated by Simpson’s method in short-axis view [22]. Therefore, we summed up the biplane end-diastolic and the end-systolic volumes, consisting of four cross-sectional areas with equal distances along the left ventricle. Stroke volume (in µL) was calculated by subtracting the end-systolic volume from the end-diastolic volume. The ejection fraction (in percentiles) was calculated as (stroke volume/end-diastolic volume) ∗ 100. The arterial function was assessed as follows: the common carotid artery was displayed longitudinally in B-mode and the pulse wave mode was applied at a proximal and distal point, 10 mm apart, to calculate the pulse-wave velocity. Data were analyzed with the Vevolab software (Visual Sonics Inc., Toronto, ON, Canada).

Noninvasive, systolic, and diastolic blood pressure were obtained by the tail-cuff method on the Coda system, with appropriate cuff sizes and restrainer for rats, and on a table heated to 37 °C to improve the detection of the tail volumes by the system (Kent Scientific Corporation, Torringtion, CT, USA) [23]. Rats were trained for at least 3 cycles and 10 cycles were recorded and averaged.

Invasive pressure was measured by Millar catheter under ketamine (100 mg/kg)/xylazine (8 mg/kg) anesthesia (intraperitoneal (i.p.)). The common carotid artery was separated from the surrounding tissue and temporarily ligated at the proximal and distal site. By arteriometry, a calibrated 1,4F pressure volume catheter was placed via the aortic arch up into the left ventricle for measurements. Continuous pressure and volume signals were recorded in real time after three stabilization phases of 5 s each. During the cardiac cycle, we recorded the maximum developed pressure (Dp max), representing the maximal contraction of the left ventricle as well as the minimum developed pressure (Dp min) [24].

### 2.3. Biochemistry

Blood was collected by tail vein puncture at baseline (*n* = 15) and after 4 weeks of adenine administration (*n* = 3–5), as well as at the end of the experiment at week 8 (*n* = 9–13). Urine was collected in metabolic cages overnight. Serum was obtained by centrifugation with 2500× *g* for 10 min at 4 °C and stored at −80 °C. Serum urea, creatinine, calcium, magnesium, protein, and phosphate were measured by routine clinical laboratory methods (Vitros 250, Ortho Clinical Diagnostics, Rochester, NY, USA). PTH and FGF23 serum levels were analyzed in serum by sandwich ELISAs according to the manufacturers’ instructions (Tecomedical, Rheinbach, Germany). Briefly, 50 µL serum was incubated on a streptavidin-coated microplate, and a biotinylated antibody was co-incubated and captured by an HRP-labeled antibody. Afterwards, the HRP substrate was incubated and stopped after 30 min, and absorbance was read at 450 nm on a microplate reader (Tecan, Männedorf, Switzerland). Concentrations were calculated by external standards. 

### 2.4. RT-PCR

RNAlater (Qiagen, Hilden, Germany) fixed tissue samples were thawed and RNA was extracted with the QIAGEN mini kit (Qiagen, Hilden, Germany). Briefly, the fixed cardiac tissue was lysed in RLT buffer (1% β-mercaptoethanol) and homogenized on a ball mill (Qiagen, Hilden, Germany). Proteinase K and DNase was purified by binding to the RNeasy membrane and eluted with ultrapure, RNase-free water.

The following primers were used: Col1 Fw5′-GACTGTCTTGCCCCAAGTTCC-3′Rv5′-GAAGGCAACAGTCGATTCACC-3′, Col III Fw5′-TCCCGAGTCGCAGTCACATA-3′Rv5′-GGGATGCAACTACCTTGGTCA-3′, Col IV Fw5′-ACATCCGGCCCTTCATTAGC-3′, Rv5′-GCACCGCCATCACCATG-3′; GAPDH: Fw 5′-AGAAGGCAGCCCTGGTAACC-3′ Rv 5′-ACAAGATGGTGAAGGTCGGTG-3′; elastin primers were from Biomol (VRPS-1839, Biomol GmbH, Hamburg, Germany). The results are represented as relative expression to relative expression of 1 million GAPDH.

### 2.5. Tissue Stainings

Trichrome Masson staining was performed on 4% PFA-fixed heart sections. The heart sections were incubated in Bouin’s solution prepared in saturated picric acid overnight at room temperature. After washing with tap water, sections were stained in Weigert’s iron hematoxylin for 5 min. After further washing steps, Biebrich Scarlet-Acid Fuchsin staining (5 min) was followed by phosphotungstic/phosphomolybdic acid. Finally, anillin blue stain (5 min) and 1% acetic acid (2 min) completed the procedure. Samples were dehydrated and mounted in Vitro-Clud (Langenbrinck, Emmendingen, Germany) [25].

For picrosirius red staining, frozen heart sections were fixed for 10 min in 4% paraformaldehyde and washed in PBS and H_2_O. Picrosirius red solution was prepared at 0.1% in picric acid at a final pH of 2. Staining was performed for 30 min, protected from light. Then, 0.01 M HCL was applied for 2 min. After washing with H_2_O and dehydrating by increasing alcohol concentrations, samples were embedded in Vitro-Clud (Langenbrinck, Emmendingen, Germany) and imaged on a Keyence BZ biorevo microscope (Keyence, Osaka, Japan) in brightfield, as was a subset in polarized light mode [26]. Picrosirius red staining enhances the natural birefringence of collagen fibers that can be visualized under cross-polarized light, leading to green- and red-yellow-emitting structures [27]. 

Von Kossa staining was performed on methanol-fixed frozen heart sections. As described earlier, sections were immersed in 1% aqueous AgNO_3_ solution for 5 min. After washing, samples were incubated in a solution of 5% NaCO_3_ and 9.25% formalin for 1 min. After a second rinse, sections were developed using sodium thiosulfate (5%) for 5 min and counterstained in 0.1% safranin-O, followed by a final rinse with tap water [28].

Immunohistochemistry was performed for collagen I (BioRad, Irvine, CA, USA), collagen III (BioRad), and elastin (Biorbyt, Cambridge, UK) after fixation with methanol of the cryosections for 1 h. A secondary horseradish peroxidase-labeled antibody was used to visualize positive staining by 3,3′-diaminobenzidine solution (Vector Laboratories, Newark, NJ, USA). Counterstaining was performed with methylgreen for 2 min. Sections were dehydrated and covered in Vitro-Clud (Langenbrinck, Emmendingen, Germany) and then imaged on a Keyence BZ biorevo microscope (Keyence, Osaka, Japan). Images of stained hearts were planimetrically quantified using ImageJ 2.9.0 1.53t. 

### 2.6. Tissue Assays

The tissue calcium content was determined in kidney, heart, aortic, and carotid artery tissue. Total calcium was mobilized by 10% formic acid overnight, followed by a colorimetrical quantification by o-cresolphthalein (Randox Laboratories, Crumlin UK). Absorbance of the supernatant was read at 550 nm on a Tecan microplate reader (Tecan, Männedorf, Switzerland) [29].

Total protein was assessed by the bicinchoninic acid method (Pierce, Thermo Fisher, Carlsbad, CA, USA) [30]. Absorbance was measured after 30 min of incubation at 37 °C at 562 nm on a microplate reader (Tecan, Männedorf, Switzerland) [30].

Hydroxyproline was analyzed after homogenization of cardiac tissue on a ball mill (Qiagen, Germany) and hydrolyzation overnight at 37 °C in 10 N NaOH, followed by neutralization by 10 N HCl. A colorimetric quantification by the reaction of oxidized hydroxyproline with 4-(dimethylamino) benzaldehyde (Sigma Aldrich, St. Louis, MO, USA) was recorded on a microplate reader (Tecan, Männedorf, Switzerland) at 560 nm [31].

### 2.7. Statistics

Shapiro Wilk and Kolmogorov–Smirnov tests were performed to assess the normality of the variables at each time point. The variables were considered as normally distributed when *p* > 0.05. If the variable was normally distributed, ANOVA was performed. For post-hoc tests, Tukey and Bonferroni corrections were applied. 

Non-normally distributed parameters were compared between the groups with Kruskal Wallis analysis. Testing for homogeneity of variances was performed. The variables have homogeneity of variances when *p* > 0.05. 

The data were analyzed with SPSS 21-25 (IBM, NewYork, NY, USA).

## 3. Results

### 3.1. Adenine-Receiving Rats Maintained Their Body Weights

Rats receiving adenine diets exhibited a lower food intake (12.7 +/− 3.1 g per day) compared with animals on a normal diet (18.1 +/− 6.8 g per day). During the study, one animal from the CKD control group 2 (in week 5) and one animal from the MgCO_3_ group 3 (in week 6) died before sacrifice. The corresponding body weights increased in the controls from baseline weights of 374 g to 538 g at week 8. In contrast, the body weight in the adenine groups remained stable between baseline (385–405 g) and week 8 (369–381 g). There were no differences in body weight or food intake between the two adenine groups.

### 3.2. Successful Induction of CKD by Adenine Diet

At week 6, serum values of creatinine, urea, phosphate, magnesium, and calcium concentrations increased significantly compared with baseline, with no differences between the two CKD groups. Serum magnesium concentrations were significantly higher in group 3, receiving the magnesium-enriched diet, compared with CKD alone (group 2) (Supplementary Table S1). Serum protein concentration was significantly lower after 4 weeks of adenine compared with the baseline (Supplementary Table S1).

At week 8, the two groups receiving adenine 2 and 3 had significantly increased serum levels of urea, creatinine, phosphate, PTH, as well as FGF23, compared with controls (Table 1). Serum concentrations of total protein and albumin levels were significantly lower in both adenine groups. There were no statistically significant differences between the two groups with adenine nephropathy.

In urine, phosphate and its fractional excretion were significantly upregulated in both adenine groups compared with controls (Table 2). Fractional excretion of calcium or magnesium remained unchanged, but the highest values were noted in the MgCO_3_ group (Table 2).

### 3.3. Preserved Left Ventricular Function by Magnesium

Systolic and diastolic blood pressure remained unchanged between all groups at the end of the experiment (Supplementary Table S2).

**Figure 2:**
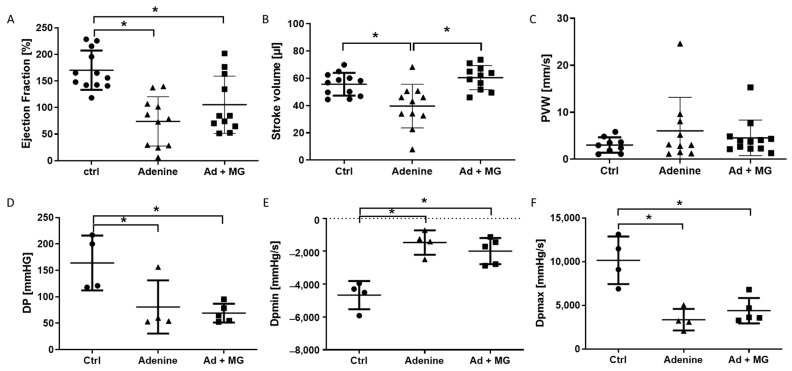
Decreased cardiac function was measured by echocardiography and Millar catheter at the end of the experiment. (**A**) Ejection fraction, (**B**) stroke volume, (**C**) pulse wave velocity (PVW) inside the common carotid artery, (**D**) left ventricular developed pressure (DP), (**E**) minimal developed pressure over time (Dpmin); and (**F**) maximal developed pressure over time (DPmax). Ad: adenine; Ctrl: control; MG: magnesium. * *p* < 0.05.

Echocardiography revealed significant changes in the cardiac function in adenine-nephropathy. Rats receiving an adenine diet showed a significant reduction in the ejection fraction (Figure 2A). Stroke volume was reduced only in the adenine group 2, whereas additional supplementation with MgCO_3_ prohibited deterioration in stroke volume, comparable to control values (Figure 2B). Pulse wave velocity of the common carotid artery remained unchanged (Figure 2C). 

In a subset of rats, a Millar catheter was placed at the end of the experiment. Both adenine groups had a significantly reduced developed pressure and maximum developed pressure inside the left ventricle (Figure 2D,F). The minimum developed pressure was higher in both adenine groups compared with controls (Figure 2E). There were no significant differences between the adenine alone group and the adenine plus magnesium group (Figure 2D–F).

### 3.4. Magnesium Increased Elastin Protein and Limited Cardiomyocyte Hypertrophy, in the Absence of Fibrosis

**Figure 3:**
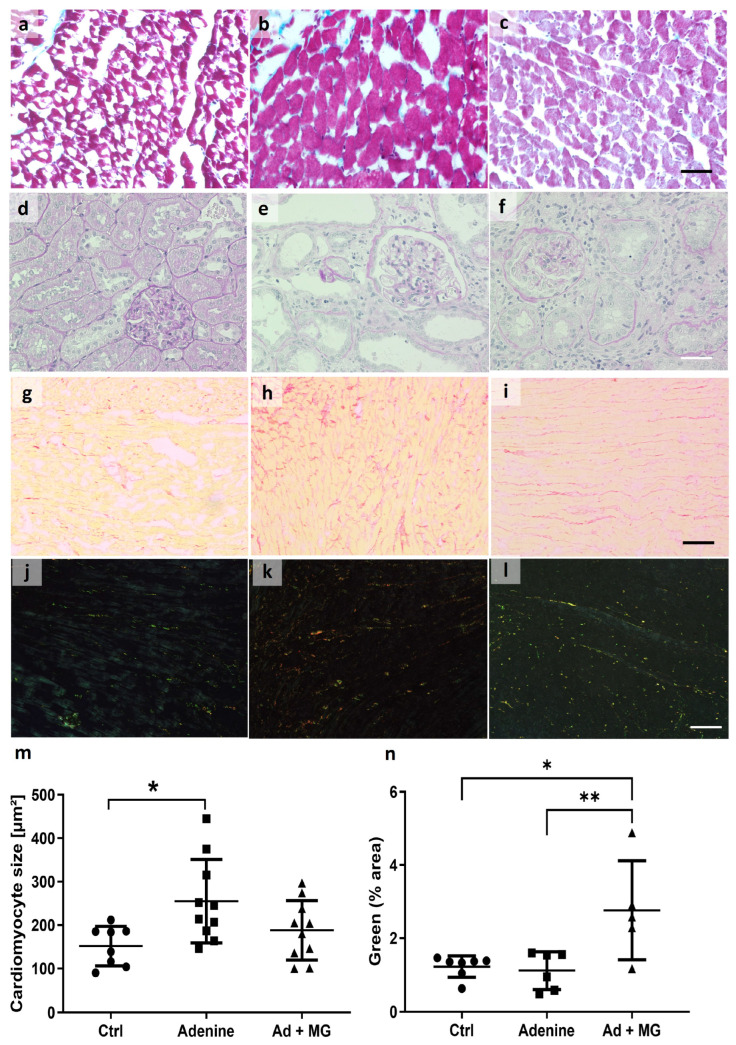
Histological analysis revealed cardiac hypertrophy but absent remarkable fibrosis in cardiac sections from adenine rats, whereas magnesium supplementation to the adenine diet increased green-emitting fibrils enhanced by polarized light after picrosirius red staining. (**a**) PAS staining in cardiac tissue from control rats; (**b**) PAS staining in cardiac tissue from adenine-fed rats; (**c**) PAS staining in cardiac tissue from adenine + magnesium-fed rats; (**d**) PAS staining in kidney tissue from control rats; (**e**) PAS staining in kidney tissue from adenine-fed rats; (**f**) PAS staining in kidney tissue from adenine + magnesium-fed rats; (**g**) picrosirius red staining in cardiac tissue from control rats, brightfield image; (**h**) sirius red staining in cardiac tissue from adenine-fed rats, brightfield image; (**i**) picrosirius red staining in cardiac tissue from adenine + magnesium fed-rats, brightfield image; (**j**) picrosirius red staining in cardiac tissue from control rats, polarized light; (**k**) picrosirius red staining in cardiac tissue from adenine-fed rats, polarized light; (**l**) picrosirius red staining in cardiac tissue from adenine + magnesium- fed rats, polarized light; (**m**) cardiomyocyte size, measured from PAS-stained sections (mean, SD); and (**n**) percentage of green-positive area in picrosirius-red-stained sections under polarized light (mean, SD). Scale = 50 µM; Ad: adenine; Ctrl: control; MG: magnesium. * *p* < 0.05, ** *p* < 0.01.

The cross-sectional area of the cardiomyocytes was measured on trichrome-stained cardiac sections. The average cardiomyocyte area was significantly increased only in the adenine group (Figure 3). The addition of magnesium carbonate to the adenine diet resulted in the maintenance of a normal cardiomyocyte size (Figure 3). 

Picrosirius red staining showed no differences between all groups. However, in an analysis of a subset of the stained sections under polarized light, increased green emission from thin collagen bundles was detected only in the magnesium-treated group 3. No differences were found in red emission intensity from thick collagen bundles (Figure 3).

**Figure 4:**
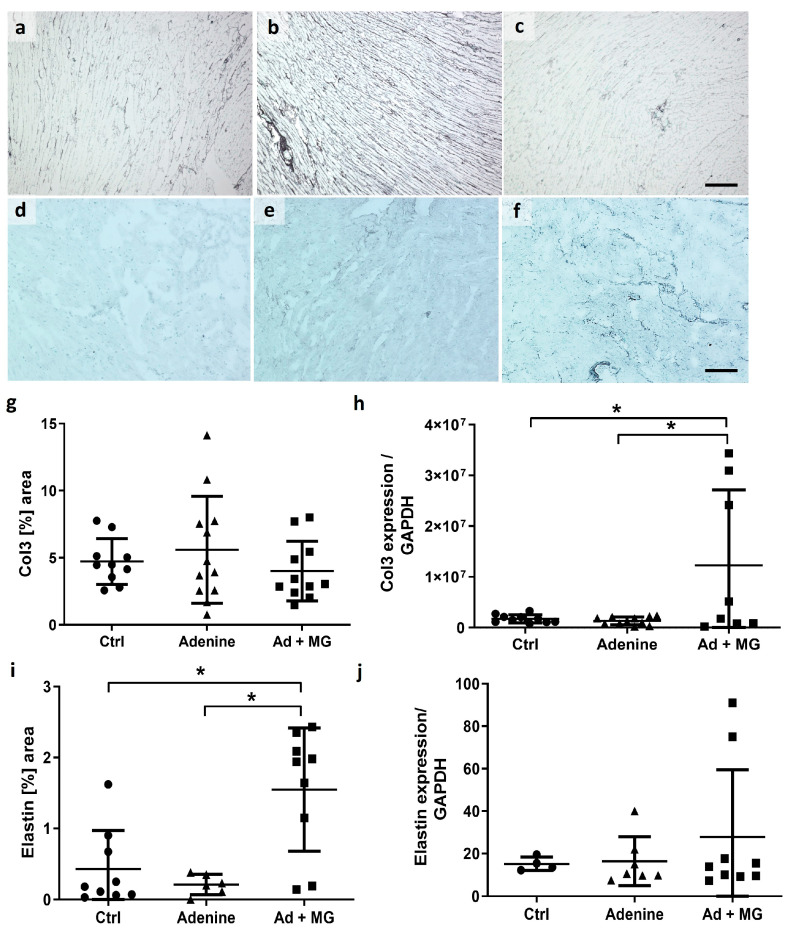
Cardiac immunohistochemistry presented an increase in elastin protein by magnesium supplementation to the adenine diet. (**a**) Collagen III staining in cardiac tissue from control rats; (**b**) collagen III staining in cardiac tissue from adenine-fed rats; (**c**) collagen III staining in cardiac tissue from adenine + magnesium-fed rats; (**d**) elastin staining in cardiac tissue from control rats; (**e**) elastin staining in cardiac tissue from adenine-fed rats; (**f**) elastin staining in cardiac tissue from adenine + magnesium-fed rats; (**g**) planimetric quantification of collagen III staining in heart tissue; (**h**) relative expression of collagen III mRNA to GAPDH, measured by semiquantitative real-time PCR; (**i**) planimetric quantification of elastin staining in hearts; (**j**) relative expression of elastin mRNA to GAPDH, measured by semiquantitative real-time PCR. Scale bar = 50 µM; Ad: adenine; Ctrl: control; MG: magnesium; *: *p* < 0.05.

Collagen III mRNA expression was significantly upregulated in hearts from the adenine plus magnesium group 3, whereas the staining of collagen III protein remained unchanged (Figure 4). In addition, the elastin-positive area on cardiac sections from the magnesium plus adenine group 3 was significantly increased compared with the adenine alone group (group 2) and controls (group 1) (Figure 4). There were no differences at the elastin mRNA level (Figure 4). Furthermore, there were no significant changes in collagen I and collagen IV mRNA expression (Supplementary Table S3).

### 3.5. Adenine Diet Induced Vascular Calcification

**Figure 5:**
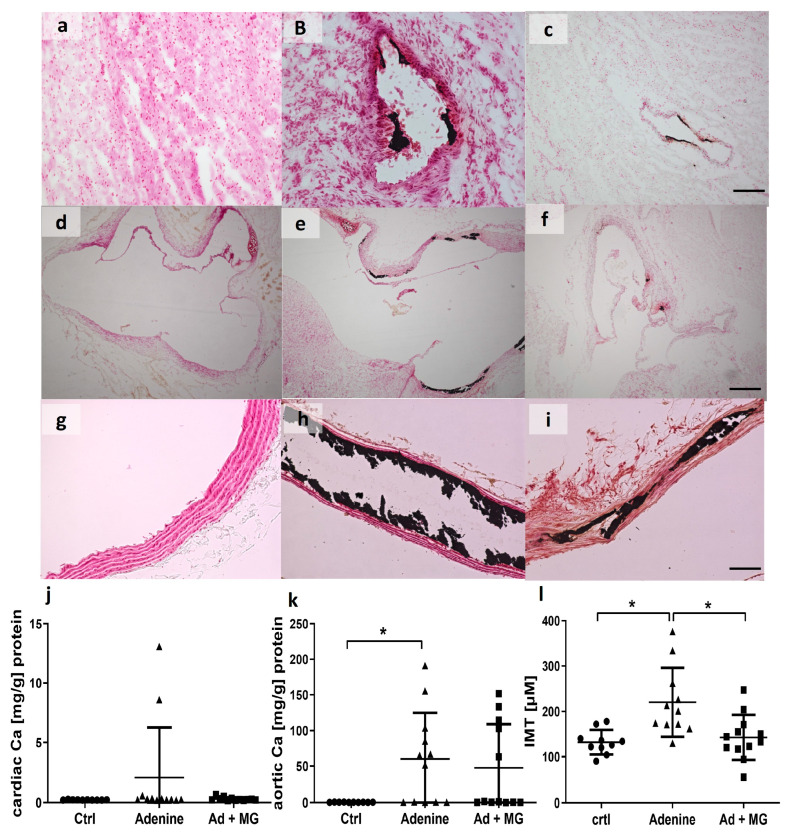
Von Kossa staining of aortas and hearts visualized an increase in soft tissue calcification with adenine diet. (**a**) Cardiac tissue from control rats; (**b**) cardiac tissue from adenine-fed rats; (**c**) cardiac tissue from adenine + magnesium-fed rats; (**d**) heart valves from control rats; (**e**) heart valves from adenine-fed rats; (**f**) heart valves from adenine + magnesium-fed rats; (**g**) aorta from control rats; (**h**) aorta from adenine-fed rats; (**i**) aorta from adenine + magnesium-fed rats; (**j**) total calcium content of cardiac tissue (mg/g) normalized to total cardiac protein; (**k**) total calcium content of aortic tissue (mg/g) normalized to total aortic protein; and (**l**) intima media thickness, measured in von Kossa-stained aortas. Scale bar = 50 µM; Ad: adenine; Ca: calcium Ctrl: control; MG: magnesium; *: *p* < 0.05.

The aortic calcium content was significantly increased in the adenine groups, but not in the magnesium group, compared with controls (Figure 5). The total cardiac calcium content failed to reach significance in the two adenine groups compared with controls. However, adenine-fed rats presented visible valve calcifications (Figure 5d–f).

The renal calcium content was significantly increased only in kidneys from the adenine group (group 2) compared with controls (group 1) (Supplementary Figure S1). We observed no significant changes in the total calcium content of the carotid arteries in the two adenine groups (groups 2 and 3) compared with controls (group 1) (Supplementary Figure S1).

A subset of calcium-positive aortas was also previously analyzed for sortilin expression [32].

## 4. Discussion

The aim of the present in vivo study was to investigate the effects of magnesium supplementation in a rodent model of CKD on cardiovascular health. CKD is often considered as similar to a state of accelerated aging [33,34]. In humans, the development of CKD is accompanied by a tremendous, life-limiting cardiovascular burden [35]. 

Here, we successfully induced CKD by feeding Wistar rats an adenine diet. Our first finding in the present animal model is that the induction of CKD in rats is accompanied by substantial cardiac changes, both at the tissue level as well as in regard to cardiac function parameters, as assessed by echocardiography and Millar catheter. The kidney–heart axis describes a clinically meaningful interaction in patients with chronic kidney disease. Facing the fact that the kidney–heart axis is a driving force in the increased morbidity and mortality in CKD patients, any animal model offering the opportunity to test potentially ameliorating interventions is of utmost clinical interest. 

Secondly, we provide experimental evidence that magnesium carbonate supplementation to the diet of rats prohibited the adenine-induced decrease in stroke volume and ejection fraction, as assessed by echocardiography. Using echocardiography allows us to identify early functional cardiac changes. Here, these underlining cardiac functional changes occurred rapidly in rats with experimental CKD, even before significant organ fibrosis was present. 

Further, we identified several microstructural changes on the tissue level, which provide strong causal explanations for the functional changes. Besides these functional changes, we found increased elastin proteins and collagen III mRNA expression, as well as increased green-light-emitting thin fiber structures in picrosirius red staining, visualized in polarized light microscopy. Hence, these experimental data point towards fundamental steps in the pathophysiology of CKD-associated cardiovascular abnormalities and, at the same time, enlighten us as to why magnesium supplementation might counterbalance them.

Elastin is the major component of elastic fibers, which are essential for proper cardiac function. Degradation of elastin is linked to numerous cardiovascular pathologies including vascular or myocardial calcification. Elastin degradation forms part of the cardiovascular risk conditions, such as diabetes mellitus and arterial hypertension, and can also be seen in aging processes [36]. Reduced elastin expression is observed post human myocardial infarction [37]. The antagonizing effect of magnesium to calcium reacting with elastin is fundamental to maintaining the elastic function [38,39]. Moreover, calcium deposits, especially microcalcifications, can induce elastin breaks [40], whereas magnesium prevents elastin degradation [38]. Our observation of higher elastin protein, compared with controls without adenine as well, points towards an effect beyond the prevention of elastin losses. Whether or not a magnesium diet itself, even in the absence of CKD, can increase the cardiac elastin content should be investigated in future studies. 

Overall, only limited effects were found regarding soft tissue calcification. As expected, aortic calcium content was increased in the adenine-fed rats. However, aortas from magnesium-supplemented rats were less calcified, whereas in other organs, no significant differences in the total calcium content could be obtained. 

To the best of our knowledge, we are the first to show a direct increase in cardiac elastin protein by magnesium supplementation in rats with adenine-induced nephropathy. The stability of elastin fibers in terms of quantity and quality is most presumably an important factor to avoid myocardial malfunction in CKD. Moreover, increased elastin-derived peptides in serum, reflecting elastin degradation, are associated with an increased all-cause mortality in CKD [41].

Next, we found green thin fibrils to be upregulated by magnesium, which is suggested to be related to collagen III [42]. Collagen fibers are synthesized in adult hearts by cardiac fibroblasts, and the total amount increases during aging. These green fibrils, visualized in polarized light after picrosirius red staining, were reported to be dominant in younger hearts, whereas the red fibrils were increasing in older hearts [43]. Whether the red and green structures reflect only the thickness of the bundles or are also associated with collagen types is debatable [26]. 

Remarkably, we indeed found an upregulation of collagen III mRNA expression with magnesium supplementation. In addition, we observed differences in the protein to mRNA level regarding both collagen III and elastin. This might indicate a time-dependent or state-of-disease-dependent effect on the regulation of these proteins. Overexpression of collagen III in injured myocardium by secretory fibroblasts was shown to prevent cardiac systolic dysfunction in ischemic cardiomyopathy in rats [44].

In addition, the beneficial effects of a magnesium-enriched diet included the mitigation of cardiac hypertrophy, measured by the cross-sectional area of the cardiomyocytes. Cardiac hypertrophy is regarded as one hallmark of uremic cardiomyopathy, besides capillary changes and fibrosis [45]. On the other hand, lower serum magnesium was found to be independently associated with left ventricular hypertrophy in dialysis patients [46]. An i.p. injection of a magnesium salt has already been shown to improve cardiac hypertrophy in a mouse model of myocardial fibrosis [47]. Even though numerous studies highlight the evidence of hypomagnesemia in CKD [1,46,48], we did not detect significant changes in the serum magnesium levels in any group. Thus, we cannot conclude on the precise impact of hypomagnesemia here.

However, our experimental data are in line with human data linking magnesium levels to specific outcome data and data about disease burden. Magnesium deficiency is common in CKD and is associated with decreased survival in observational studies [25]. An improvement in cardiovascular health by magnesium supplementation has already been reported in various human cardiovascular diseases [2,3,4,5,6,7,8,9,10,11,49]. Magnesium supplementation via oral administration thus targets two relevant disease conditions with a high prevalence in CKD patients: hyperphosphatemia and hypomagnesemia. As such, magnesium-based phosphate binders might be of specific interest as they combine both on-target (phosphate-lowering) and off-target (magnesium-increasing) effects. 

Taken together, our experimental data point towards cardioprotective effects of dietary magnesium supplementation in a rodent model of experimental nephropathy. A strength of our study is that we can show a plausible link between echocardiography and microstructural findings.

One limitation of the present study is that we can only provide limited mechanistic insights into the role of magnesium in the prevention of cardiovascular disease in the setting of experimental uremia. Secondly, we did not observe any functional changes induced by magnesium measured by the Millar catheter. We assume the changes in elastin and collagen III patterns with magnesium supplementation promote the contractility of the cardiac tissue, which may partially explain the restored stroke volumes and ejection fractions. However, whether or not our observations in the rodent model also apply to human CKD requires interventional data from appropriately designed randomized, controlled studies.

## 5. Conclusions

Magnesium supplementation exerted beneficial effects protecting the myocardium in rats with experimental CKD. We identified magnesium deficiency as a potentially reversible cause of altered cardiovascular structure and impaired cardiovascular function. 

## Figures and Tables

**Table 1:** Serum biochemistry at the end of the experiment, week 8, highlighting a successful induction of CKD by adenine diet. ALP: alkaline phosphatase; Ca x P: calcium phosphate product; PTH: parathyroid hormone; FGF23: fibroblast growth factor 23.

	Control	Adenine	Adenine +MgCO_3_
Creatinine, mg/dL, median (IQR)	0.4 (0.32–0.49)	1.7 (1.3–5.1) *	1.9 (1.1–5.4) *
Urea, mg/dL, median (IQR)	1.5 (13.2–18.5)	66.1 (51.8–212.9) *	49.6 (35.6–172.5) *
Phosphate, mg/dL, median (IQR)	6.6 (6.2–8.1)	15.4 (10.6–23.8) *	10.9 (8.3–23.8) *
Calcium, mg/dL, median (IQR)	11.4 (11.1–11.7)	11.8 (11–12.7)	11.7 (11.2–12)
Ca × P, mg^2^/dL^2^, median (IQR)	74 (70.6–97.3)	191.8 (121–260.9) *	127.7 (91.1–300.5) *
Magnesium, mg/dL, median (IQR)	2.9 (2.7–2.9)	2.9 (2.7–3.4)	2.9 (2.7–4.8)
ALP, UI/L, mean (SD)	91.7 ± 16.4	97.54 ± 34.72	107.17 ± 43.25
PTH, pg/mL, mean (SD)	1700 ± 918	5338 ± 4525 *	4133 ± 1396 *
FGF23, ng/mL, median (IQR)	0.34 (0.28–0.41)	9.84 (2.72–30.95) *	24.1 (1.15–286.75) *
Protein, g/dL, median (IQR)	5.8 (5.6–6.02)	5.4 (5–5.8) *	5.2 (5–5.5) *
Albumin, g/dL, median (IQR)	2.9 (2.8–3.2)	2.6 (2.4–2.8) *	2.5 (2.3–2.8) *

* Significant to control (group 1).

**Table 2:** Urine biochemistry at the end of the experiment, week 8, highlighting a successful induction of CKD by adenine diet (FECa: fractional excretion of calcium; FEMg: fractional excretion of magnesium; FEP: fractoinal excretion of phosphate).

	Control	Adenine	Adenine + MgCO_3_
Phosphate mg/24 h, mean (SD)	12.89 ± 4.15	48.10 ± 12.65 *	43.03 ± 12.47 *
FEP %, median (IQR)	5.1 (4.6–7.8)	67 (60.6–80.7) *	69.4 (52.3–85.5) *
Magnesium mg/24 h, median (IQR)	2.9 (2.1–4.5)	3.3 (2.5–4.7)	4.36 (2.6–5.9)
FEMg %, median (IQR)	13.2 (3.1–30.9)	16.9 (3.5–44.6)	28.6 (21.9–53.9)
Calcium mg/24 h, mean (SD)	2.62 ± 2.07	3.16 ± 1.75	5.23 ± 2.32
FECa %, median (IQR)	2 (0.35–10.2)	3.27 (0.29–13.3)	8.5 (6.1–24)
Protein mg/24 h, median (IQR)	12.1 (10.3–16)	11.4 (9.3–16.1)	12.9 (9.7–18.2)

* Significant to control (group 1).

## Data Availability

Data sharing not applicable.

## Supplementary Materials

The following supporting information can be downloaded at: https://www.mdpi.com/article/10.3390/nu15061303/s1, Figure S1: Calcium contents in kidney and carotid artery. (A) Total calcium content of kidney tissue [mg/g] normalized to total renal protein, (B) Total calcium content of carotid artery tissue [mg/g] normalized to total carotid artery protein; Ad: Adenine; Ca: Calcium; Ctrl: Control; MG: Magnesium; *: *p* < 0.05; Table S1: Serum biochemistry (A) At baseline (B) At week 6. * Significant versus baseline. # Significant versus adenine; Table S2: Blood pressure at baseline and at week 8; Table S3: Relative expression of collagen I and collagen IV mRNA to relative expression of GAPDH mRNA.

## References

[1] Azem R., Daou R., Bassil E., Anvari E.M., Taliercio J.J., Arrigain S., Schold J.D., Vachharajani T., Nally J., Na Khoul G.N. (2020). Serum magnesium, mortality and disease progression in chronic kidney disease. BMC Nephrol..

[2] Reffelmann T., Ittermann T., Dorr M., Volzke H., Reinthaler M., Petersmann A., Felix S.B. (2011). Low serum magnesium concentrations predict cardiovascular and all-cause mortality. Atherosclerosis.

[3] Bouida W., Beltaief K., Msolli M.A., Azaiez N., Ben Soltane H., Sekma A., Trabelsi I., Boubaker H., Grissa M.H., Methemem M. (2019). Low-dose Magnesium Sulfate Versus High Dose in the Early Management of Rapid Atrial Fibrillation: Randomized Controlled Double-blind Study (LOMAGHI Study). Acad. Emerg. Med..

[4] Davey M.J., Teubner D. (2005). A randomized controlled trial of magnesium sulfate, in addition to usual care, for rate control in atrial fibrillation. Ann. Emerg. Med..

[5] Wang A. (2012). Efficacy of class III antiarrhythmics and magnesium combination therapy for atrial fibrillation. Pharm. Pract..

[6] Salaminia S., Sayehmiri F., Angha P., Sayehmiri K., Motedayen M. (2018). Evaluating the effect of magnesium supplementation and cardiac arrhythmias after acute coronary syndrome: A systematic review and meta-analysis. BMC Cardiovasc. Disord..

[7] Pokan R., Hofmann P., von Duvillard S.P., Smekal G., Wonisch M., Lettner K., Schmid P., Shechter M., Silver B., Bachl N. (2006). Oral magnesium therapy, exercise heart rate, exercise tolerance, and myocardial function in coronary artery disease patients. Br. J. Sports Med..

[8] Almoznino-Sarafian D., Berman S., Mor A., Shteinshnaider M., Gorelik O., Tzur I., Alon I., Modai D., Cohen N. (2007). Magnesium and C-reactive protein in heart failure: An anti-inflammatory effect of magnesium administration?. Eur. J. Nutr..

[9] Cunha A.R., D’El-Rei J., Medeiros F., Umbelino B., Oigman W., Touyz R.M., Neves M.F. (2017). Oral magnesium supplementation improves endothelial function and attenuates subclinical atherosclerosis in thiazide-treated hypertensive women. J. Hypertens..

[10] Mathers T.W., Beckstrand R.L. (2009). Oral magnesium supplementation in adults with coronary heart disease or coronary heart disease risk. J. Am. Acad. Nurse Pract..

[11] Liu M., Dudley S.C. (2020). Magnesium, Oxidative Stress, Inflammation, and Cardiovascular Disease. Antioxidants.

[12] Baker W.L. (2017). Treating arrhythmias with adjunctive magnesium: Identifying future research directions. Eur. Heart J. Cardiovasc. Pharmacother..

[13] Floege J. (2015). Magnesium in CKD: More than a calcification inhibitor?. J. Nephrol..

[14] Lioufas N.M., Pascoe E.M., Hawley C.M., Elder G.J., Badve S.V., Block G.A., Johnson D.W., Toussaint N.D. (2022). Systematic Review and Meta-Analyses of the Effects of Phosphate-Lowering Agents in Nondialysis CKD. J. Am. Soc. Nephrol..

[15] Ruospo M., Palmer S.C., Natale P., Craig J.C., Vecchio M., Elder G.J., Strippoli G.F. (2018). Phosphate binders for preventing and treating chronic kidney disease-mineral and bone disorder (CKD-MBD). Cochrane Database Syst. Rev..

[16] Doshi S.M., Wish J.B. (2022). Past, Present, and Future of Phosphate Management. Kidney Int. Rep..

[17] Leenders N.H.J., Vervloet M.G. (2019). Magnesium: A Magic Bullet for Cardiovascular Disease in Chronic Kidney Disease?. Nutrients.

[18] Sakaguchi Y. (2022). The emerging role of magnesium in CKD. Clin. Exp. Nephrol..

[19] Diwan V., Brown L., Gobe G.C. (2018). Adenine-induced chronic kidney disease in rats. Nephrology (Carlton).

[20] Klinkhammer B.M., Djudjaj S., Kunter U., Palsson R., Edvardsson V.O., Wiech T., Thorsteinsdottir M., Hardarson S., Foresto-Neto O., Mulay S.R. (2020). Cellular and Molecular Mechanisms of Kidney Injury in 2,8-Dihydroxyadenine Nephropathy. J. Am. Soc. Nephrol..

[21] Kaesler N., Magdeleyns E., Herfs M., Schettgen T., Brandenburg V., Fliser D., Vermeer C., Floege J., Schlieper G., Kruger T. (2014). Impaired vitamin K recycling in uremia is rescued by vitamin K supplementation. Kidney Int..

[22] Arias T., Chen J., Fayad Z.A., Fuster V., Hajjar R.J., Chemaly E.R. (2013). Comparison of echocardiographic measurements of left ventricular volumes to full volume magnetic resonance imaging in normal and diseased rats. J. Am. Soc. Echocardiogr..

[23] Daugherty A., Rateri D., Hong L., Balakrishnan A. (2009). Measuring blood pressure in mice using volume pressure recording, a tail-cuff method. J. Vis. Exp..

[24] Pacher P., Nagayama T., Mukhopadhyay P., Batkai S., Kass D.A. (2008). Measurement of cardiac function using pressure-volume conductance catheter technique in mice and rats. Nat. Protoc..

[25] Van De Vlekkert D., Machado E., d’Azzo A. (2020). Analysis of Generalized Fibrosis in Mouse Tissue Sections with Masson's Trichrome Staining. Bio. Protoc..

[26] Lattouf R., Younes R., Lutomski D., Naaman N., Godeau G., Senni K., Changotade S. (2014). Picrosirius red staining: A useful tool to appraise collagen networks in normal and pathological tissues. J. Histochem. Cytochem..

[27] Junqueira L.C., Bignolas G., Brentani R.R. (1979). Picrosirius staining plus polarization microscopy, a specific method for collagen detection in tissue sections. Histochem. J..

[28] Kaesler N., Goettsch C., Weis D., Schurgers L., Hellmann B., Floege J., Kramann R. (2020). Magnesium but not nicotinamide prevents vascular calcification in experimental uraemia. Nephrol. Dial. Transplant..

[29] Morin L.G. (1974). Direct colorimetric determination of serum calcium with o-cresolphthalein complexon. Am. J. Clin. Pathol..

[30] Walker J.M. (1994). The bicinchoninic acid (BCA) assay for protein quantitation. Methods Mol. Biol..

[31] Reddy G.K., Enwemeka C.S. (1996). A simplified method for the analysis of hydroxyproline in biological tissues. Clin. Biochem..

[32] Jankowski V., Saritas T., Kjolby M., Hermann J., Speer T., Himmelsbach A., Mahr K., Heuschkel M.A., Schunk S.J., Thirup S. (2022). Carbamylated sortilin associates with cardiovascular calcification in patients with chronic kidney disease. Kidney Int..

[33] Kooman J.P., Kotanko P., Schols A.M., Shiels P.G., Stenvinkel P. (2014). Chronic kidney disease and premature ageing. Nat. Rev. Nephrol..

[34] Ebert T., Pawelzik S.C., Witasp A., Arefin S., Hobson S., Kublickiene K., Shiels P.G., Back M., Stenvinkel P. (2020). Inflammation and Premature Ageing in Chronic Kidney Disease. Toxins.

[35] Jankowski J., Floege J., Fliser D., Bohm M., Marx N. (2021). Cardiovascular Disease in Chronic Kidney Disease Pathophysiological Insights and Therapeutic Options. Circulation.

[36] Cocciolone A.J., Hawes J.Z., Staiculescu M.C., Johnson E.O., Murshed M., Wagenseil J.E. (2018). Elastin, arterial mechanics, and cardiovascular disease. Am J. Physiol. Heart Circ. Physiol..

[37] Novitskaya T., Nishat S., Covarrubias R., Wheeler D.G., Chepurko E., Bermeo-Blanco O., Xu Z., Baer B., He H., Moore S.N. (2022). Ectonucleoside triphosphate diphosphohydrolase-1 (CD39) impacts TGF-beta1 responses: Insights into cardiac fibrosis and function following myocardial infarction. Am. J. Physiol. Heart Circ. Physiol..

[38] Muller W., Iffland R., Firsching R. (1993). Relationship between magnesium and elastic fibres. Magnes. Res..

[39] Muller W., Firsching R. (1991). Demonstration of elastic fibres with reagents for detection of magnesium. J. Anat..

[40] Basalyga D.M., Simionescu D.T., Xiong W., Baxter B.T., Starcher B.C., Vyavahare N.R. (2004). Elastin degradation and calcification in an abdominal aorta injury model: Role of matrix metalloproteinases. Circulation.

[41] Smith E.R., Tomlinson L.A., Ford M.L., McMahon L.P., Rajkumar C., Holt S.G. (2012). Elastin degradation is associated with progressive aortic stiffening and all-cause mortality in predialysis chronic kidney disease. Hypertension.

[42] Junqueira L.C., Montes G.S., Sanchez E.M. (1982). The influence of tissue section thickness on the study of collagen by the Picrosirius-polarization method. Histochemistry.

[43] Debessa C.R.G., Maifrino L.B.M., de Souza R.R. (2001). Age related changes of the collagen network of the human heart. Mech. Ageing Dev..

[44] Uchinaka A., Yoshida M., Tanaka K., Hamada Y., Mori S., Maeno Y., Miyagawa S., Sawa Y., Nagata K., Yamamoto H. (2018). Overexpression of collagen type III in injured myocardium prevents cardiac systolic dysfunction by changing the balance of collagen distribution. J. Thorac. Cardiovasc. Surg..

[45] Kaesler N., Babler A., Floege J., Kramann R. (2020). Cardiac Remodeling in Chronic Kidney Disease. Toxins.

[46] Balafa O., Dounousi E., Giannikouris I., Petrakis I., Georgoulidou A., Karassavidou D., Kokalis A., Stauroulopoulos A., Theodoridis M., Oikonomidis I. (2022). Lower serum magnesium is a predictor of left ventricular hypertrophy in patients on dialysis. Int. Urol. Nephrol..

[47] Ma D., Zhang J., Zhang Y., Zhang X., Han X., Song T., Zhang Y., Chu L. (2018). Inhibition of myocardial hypertrophy by magnesium isoglycyrrhizinate through the TLR4/NF-kappaB signaling pathway in mice. Int. Immunopharmacol..

[48] Cunningham J., Rodriguez M., Messa P. (2012). Magnesium in chronic kidney disease Stages 3 and 4 and in dialysis patients. Clin. Kidney J..

[49] Karimi M., Mohammadi F., Behmanesh F., Samani S.M., Borzouee M., Amoozgar H., Haghpanah S. (2010). Effect of combination therapy of hydroxyurea with l-carnitine and magnesium chloride on hematologic parameters and cardiac function of patients with beta-thalassemia intermedia. Eur. J. Haematol..

