# Supplementary material for: Magnesium Improves Cardiac Function in Experimental Uremia by Altering Cardiac Elastin Protein Content
Source: Nutrients. 2023 Mar 7;15(6):1303. doi: 10.3390/nu15061303 (PMC10056411; doi:10.3390/nu15061303)
Supplement: Supplementary file 1 [file nutrients-15-01303-s001.zip › nutrients-2209784-supplementary.pdf]

Supplementary Materials:

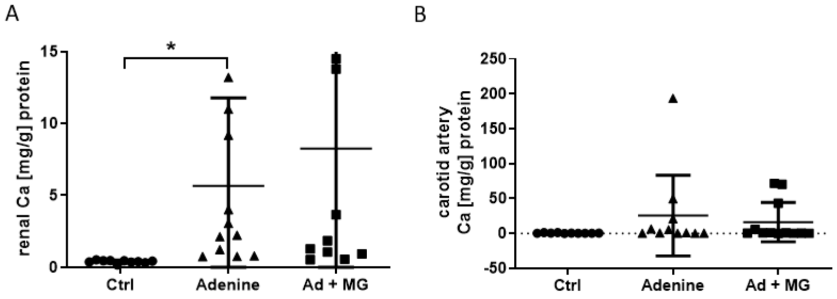

Figure S1. Calcium contents in kidney and carotid artery. (A) Total calcium content of kidney tissue [mg/g] normalized to total renal protein; (B) Total calcium content of carotid artery tissue [mg/g] normalized to total carotid artery protein; Ad: Adenine; Ca: Calcium; Ctrl: Control; MG: Magnesium; *: $p < 0.05$.

Table S1. Serum biochemistry.

**A: At baseline**		
**Parameter at baseline**	**Mean**	**SD**
Urea (mg/dL)	18.99	3.43
Creatinine (mg/dL)	0.36	0.08
Phosphate (mg/dL)	8.24	0.68
Magnesium (mg/dL)	2.27	0.25
Calcium (mg/dL)	10.44	0.27
Protein (mg/dL)	6.52	0.43
**B: At week 6**		
**Parameter at week 6**	**Adenine**	**Adenine + $MgCO_3$**
Creatinine, mg/dL (mean, SD)	3.11 ± 1.55*	3.29 ± 0.7*
Urea, mg/dL (mean, SD)	46.19 ± 18*	49.11 ± 18.61*
Phosphate, mg/dL (mean, SD)	11.91 ± 2.92*	11.51 ± 3.45*
Calcium, mg/dL (mean, SD)	11.66 ± 0.7 *	11.96 ± 0.62*
Magnesium, mg/dL (mean, SD)	4 ± 1.1*	5.14 ± 0.99 *#
Protein, mg/dL (mean, SD)	5.35 ± 0.24*	5.51 ± 0.28*

* Significant versus baseline. # Significant versus adenine.

Table S2. Blood pressure at baseline and at week 8.

**Blood pressure**	**Systole**		**Diastole**	
	**Mean**	**SD**	**Mean**	**SD**
Baseline	126	13	88	16
Control, week 8	133	21	90	21
Adenine, week 8	131	23	81	17
Adenine + $MgCO_3$, week 8	136	17	90	18

Table S3: Relative expression of collagen I and collagen IV mRNA to relative expression of GAPDH mRNA.

	**Ctrl**	**Adenine**	**Adenine + $MgCO_3$**
Collagen I, mean (SD)	1,356,287 (867,270)	1,001,005 (607,969)	2,070,345 (2,845,104)
Collagen IV, mean (SD)	49,084 (44,122)	269,731 (736052)	70,957 (64,635)
